# Editorial: paramedic careers and research – why it matters and how to start

**DOI:** 10.29045/14784726.2026.6.11.1.1

**Published:** 2026-06-01

**Authors:** Larissa Prothero, Helen Pocock, Caitlin Wilson, Julia Wolfe

**Affiliations:** East of England Ambulance Service NHS Trust ORCID iD: https://orcid.org/0000-0002-5440-8429; South Central Ambulance Service NHS Foundation Trust ORCID iD: https://orcid.org/0000-0001-7648-5313; Yorkshire Ambulance Service NHS Trust; University of Hertfordshire ORCID iD: https://orcid.org/0000-0002-9854-4289; Northern Ireland Ambulance Service Health and Social Care Trust ORCID iD: https://orcid.org/0000-0002-3104-8360

**Keywords:** capability, capacity, career, paramedic, research

Paramedics today have a variety of career pathways to explore, including clinical practice, leadership, education and research (Royal College of Paramedics, 2024). While research may not always be the most immediately obvious option, it is increasingly recognised as a legitimate career trajectory ‒ either as a dedicated pathway or often combined with other roles, such as clinical or educational practice. Research is one of the four pillars of professional practice, contributing to the evidence that underpins patient care, service development and the ongoing growth of the profession (Whitley & Wilson, 2022).

Choosing to engage in research allows clinicians to follow their curiosity to answer questions that arise from everyday practice and to contribute to new knowledge that can influence care beyond their immediate setting. It offers opportunities for autonomy in selecting topics and shaping studies, while also providing professional satisfaction through achieving tangible outputs, such as publications, service improvements or changes in clinical guidelines. Perhaps most importantly, research has the potential for lasting impact at multiple levels, from improving patient outcomes to informing policy and practice. While these aspects are not exclusive to research careers, they highlight why pursuing research can be a meaningful complement or focus within a paramedic career (Whitley & Wilson, 2022).

Despite its value, many paramedics experience barriers when considering a research pathway. Studies across allied health professions highlight challenges including limited capacity, lack of protected time, gaps in research knowledge and the absence of mentors or supportive networks (Thamm et al., 2026; Trusson et al., 2019). Within paramedicine specifically, qualitative research has demonstrated that even clinicians motivated to engage in research often struggle with these practical and structural hurdles (McClelland et al., 2023).

If research is to become a more widely embraced pillar of practice, it helps to start with reflection. A practical first step for individuals is to consider their current position in terms of research skills. Using a framework discussed in the next section, they can identify their position in their research journey and actionable steps to engage meaningfully with research, whether as a sole focus or as part of a portfolio career.

## Measuring your research capability

Research *capability* describes the theoretical and practical skills necessary to become research active, whereas research *capacity* is the ability to conduct research (Whitehouse et al., 2022). For many clinicians, identifying which skills they need and assessing their current level can feel challenging, particularly when shifting focus from a purely clinical role towards research. Various tools are available to map academic research skills, such as the Vitae Researcher Development Framework (Vitae, 2025), but these lack the relevance and accessibility required by non-academic health and care professionals.

The NHS England Multi-professional Practice-based Research Capabilities Framework describes a set of core capabilities laid out in eight domains relevant to all health and care professionals outside of medicine and dentistry (NHS England, 2023). Each domain includes four levels of capability, which correspond to the four levels of professional practice: Entry, Enhanced, Advanced and Consultant. This framework allows individuals to benchmark their current skill set ‒ as inspiration for progress ‒ and to plan and map their progress. The eight domains span the research function, thereby giving users a broad research skill set:

- Research-related career growth
- Planning and designing research
- Delivering research
- Knowledge mobilisation and research implementation
- Networking and collaborating in research
- Supporting research-related development in others
- Leading and managing research projects and teams
- Strategic leadership in research and knowledge mobilisation.

Most capabilities are described and developed across the four levels. For example, *Entry*: Analyse research evidence relevant to own practice → *Enhanced*: Contribute to local evidence-based guidance → *Advanced*: Contribute to developments in practice at a service level informed by research findings → *Consultant*: Contribute to national/international guidance.

The usefulness of a framework to individual practitioners will be limited by their context; a lack of strategic enablers, such as an organisational research culture, can stifle progress (Watson & Kamalakannan, 2025). Development of research capability must be matched with capacity building for research activity to flourish.

## Building your research capability

Increasing individual research capability can feel daunting even after undertaking a mapping exercise; however, small, deliberate research-related activities can build research confidence and sustain motivation by offering early successes. Although increasing theoretical research knowledge is a good starting point to building a strong research foundation, the development of practical research skills is often best achieved under the guidance and mentorship of experienced researchers, who can assist with the translation of theory into practice.

### Developing theoretical knowledge

Various online resources are available to support the development of research knowledge. In the UK, platforms such as National Institute for Health and Care Research (NIHR) Learn (NHS Health Research Authority, 2024) and NHS England eLearning for Healthcare (NHS England, 2026) offer a variety of structured research learning opportunities with a practical focus. Topic modules include research methodology, research study governance and delivery, as well as research leadership. Undertaking such courses and participating in self-directed continuous professional development using specialist resources (e.g. research textbooks and published literature), participating in journal clubs, contributing as a peer reviewer for academic journals and attending research conferences all provide accessible and effective means of enhancing research knowledge and skills. Whether delivering a presentation (oral or poster) or attending as a delegate, conference attendance is considered beneficial for exposure to current and future research activities and ideas, personal development opportunities and networking with peers and subject experts. There are now numerous national and international research conferences that support ambulance and emergency medicine research.

The Royal College of Paramedics Research Centre (Royal College of Paramedics, 2026) and Community for Allied Health Professions Research (CAHPR) also provide access to capability- and capacity-building resources. They offer research podcasts, drop-in clinics, e-learning resources, study participation opportunities, research directories and signposting to research funding opportunities, all of which support research growth and collaboration for the paramedic profession. Beyond these organisations, others, such as the Irish Paramedicine Education and Research Network, the Australasian College of Paramedicine and the McNally Project, provide similar support for the advancement of pre-hospital research in the ambulance setting.

### Developing practical research skills

Whether a single research idea warrants development or an individual is considered to be an early career researcher, it is recognised that research mentors and facilitators play vital roles in enabling individuals to develop their research capacity (Cooke et al., 2018). This support can be at local and peer level, or part of a formal arrangement with a higher education institute (HEI) providing the research training programme. The NIHR includes mentorship within its principles for nurses, midwives and allied health professionals undertaking clinical academic training (National Institute for Health and Care Research (NIHR), 2021). The range of clinical academic development opportunities for allied health professionals (including paramedics) is expanding world-wide, from internships and undergraduate and postgraduate degrees to post-doctoral opportunities and professorships. In the UK, key funding and support for personal research training and academic career development can be found typically via the NIHR Academy, UK Research and Innovation Research Councils, HEIs and charitable organisations.

Beyond the HEI environment and while continuing clinical practice, paramedics (and other ambulance staff) may also seek guidance and support from their local ambulance service research delivery team. All UK ambulance services have a dedicated research team that actively facilitates and delivers research (National Ambulance Research Steering Group, 2024). These teams provide guidance on key research processes, such as research conduct, funding opportunities, study design and feasibility, research governance and ethical and regulatory approvals (Sanderson & Prothero, 2025). When seeking to undertake research in conjunction with an NHS organisation, whether it involves ambulance staff or patients as participants, early engagement with the research team is key for the successful development and delivery of any study.

For paramedics and other ambulance staff who do not wish to consider further education and research as a career pathway, there are other ways to contribute to ambulance-based research and influence practice change. High-quality service evaluations and audits can provide initial evidence and the foundations upon which new research studies are developed. Active engagement and involvement in studies can offer unique opportunities to contribute and translate research knowledge into practice. Recruiting and consenting patients into a clinical trial can provide meaningful insights into research and new clinical experiences, while contributing to personal professional development. Being involved as ‘an expert by lived experience’ in a study team or trial management team can ensure that the voices of both the ambulance clinician and the patient are included throughout the course of the research. Furthermore, being a study participant not only means directly contributing to a new evidence base but also gaining first-hand practical experiences of research.

To conclude, for paramedics and other ambulance-based healthcare professionals who wish to pursue research as part of their professional career, there is an array of guidance and opportunities for individual research capability and capacity building. Supporting these individuals to pursue this pillar of practice will result in the development of a sustainable ambulance research workforce at all practice levels.

## Conflict of interest

LP is on the Editorial Board for the *British Paramedic Journal*; CW is an Associate Editor for the *British Paramedic Journal*.

## Statement of generative AI in scientific writing

The authors did not use a generative artificial intelligence (AI) tool or service to assist with preparation or editing of this work.
